# Supplementary material for: Modeling glioblastoma heterogeneity as a dynamic network of cell states
Source: Mol Syst Biol. 2021 Sep 16;17(9):e10105. doi: 10.15252/msb.202010105 (PMC8444284; doi:10.15252/msb.202010105)
Supplement: Supplementary file 6 — Source Data for Figure 5 [file MSB-17-e10105-s004.zip › Figure5A_sourcedata/GSEA_3071/nosigdb_stateVI.GseaPreranked.1622795169056/RICHARDS_ZHONG_NATURE_2018_ASTROCYTES_UPREG.html]

Details for gene set RICHARDS\_ZHONG\_NATURE\_2018\_ASTROCYTES\_UPREG[GSEA]

|  || Dataset | state63071 |
| Phenotype | NoPhenotypeAvailable |
| Upregulated in class | na\_neg |
| GeneSet | RICHARDS\_ZHONG\_NATURE\_2018\_ASTROCYTES\_UPREG |
| Enrichment Score (ES) | -0.5121277 |
| Normalized Enrichment Score (NES) | -2.6556225 |
| Nominal p-value | 0.0 |
| FDR q-value | 0.0 |
| FWER p-Value | 0.0 |
Table: GSEA Results Summary

  

Fig 1: Enrichment plot: RICHARDS\_ZHONG\_NATURE\_2018\_ASTROCYTES\_UPREG      
 Profile of the Running ES Score & Positions of GeneSet Members on the Rank Ordered List

  

| PROBE | GENE SYMBOL | GENE\_TITLE | RANK IN GENE LIST | RANK METRIC SCORE | RUNNING ES | CORE ENRICHMENT || 1 | ALCAM |  |  | 7 | 1.185 | 0.0086 | No |
| 2 | MT1E |  |  | 10 | 1.128 | 0.0180 | No |
| 3 | RGCC |  |  | 17 | 1.065 | 0.0258 | No |
| 4 | F3 |  |  | 26 | 0.988 | 0.0323 | No |
| 5 | MT2A |  |  | 58 | 0.859 | 0.0311 | No |
| 6 | HOPX |  |  | 89 | 0.761 | 0.0293 | No |
| 7 | MGLL |  |  | 190 | 0.609 | 0.0061 | No |
| 8 | ADORA2B |  |  | 192 | 0.608 | 0.0113 | No |
| 9 | RAB34 |  |  | 220 | 0.589 | 0.0088 | No |
| 10 | RGS20 |  |  | 259 | 0.561 | 0.0029 | No |
| 11 | RHOC |  |  | 277 | 0.551 | 0.0029 | No |
| 12 | ZFP36L2 |  |  | 284 | 0.547 | 0.0061 | No |
| 13 | CDC42EP1 |  |  | 318 | 0.529 | 0.0014 | No |
| 14 | JUN |  |  | 321 | 0.527 | 0.0055 | No |
| 15 | HSPB1 |  |  | 322 | 0.527 | 0.0102 | No |
| 16 | LGALS1 |  |  | 323 | 0.526 | 0.0149 | No |
| 17 | CCDC80 |  |  | 343 | 0.514 | 0.0140 | No |
| 18 | PLAGL1 |  |  | 400 | 0.490 | 0.0024 | No |
| 19 | GLUD1 |  |  | 433 | 0.476 | -0.0025 | No |
| 20 | GPX3 |  |  | 493 | 0.457 | -0.0154 | No |
| 21 | SLC3A2 |  |  | 559 | 0.437 | -0.0301 | No |
| 22 | DIO2 |  |  | 567 | 0.434 | -0.0282 | No |
| 23 | IGFBP4 |  |  | 571 | 0.433 | -0.0252 | No |
| 24 | PHACTR2 |  |  | 580 | 0.431 | -0.0236 | No |
| 25 | SERPINB6 |  |  | 583 | 0.430 | -0.0204 | No |
| 26 | EFHD2 |  |  | 598 | 0.425 | -0.0206 | No |
| 27 | ALPL |  |  | 613 | 0.422 | -0.0208 | No |
| 28 | RCN1 |  |  | 641 | 0.413 | -0.0249 | No |
| 29 | CTSL |  |  | 767 | 0.383 | -0.0573 | No |
| 30 | PLIN3 |  |  | 781 | 0.380 | -0.0576 | No |
| 31 | NT5E |  |  | 787 | 0.379 | -0.0556 | No |
| 32 | VIM |  |  | 897 | 0.361 | -0.0836 | No |
| 33 | YIF1A |  |  | 964 | 0.351 | -0.0994 | No |
| 34 | PDLIM5 |  |  | 1062 | 0.335 | -0.1242 | No |
| 35 | TRIM47 |  |  | 1069 | 0.334 | -0.1229 | No |
| 36 | GNG12 |  |  | 1178 | 0.319 | -0.1510 | No |
| 37 | TFPI |  |  | 1205 | 0.315 | -0.1556 | No |
| 38 | TIMP3 |  |  | 1290 | 0.305 | -0.1769 | No |
| 39 | S1PR1 |  |  | 1306 | 0.302 | -0.1785 | No |
| 40 | LIFR |  |  | 1567 | 0.272 | -0.2505 | No |
| 41 | LRRC17 |  |  | 1593 | 0.269 | -0.2553 | No |
| 42 | SDC3 |  |  | 1818 | -0.252 | -0.3171 | No |
| 43 | NOTCH3 |  |  | 1872 | -0.260 | -0.3300 | No |
| 44 | TOR1AIP1 |  |  | 1985 | -0.276 | -0.3596 | No |
| 45 | LRRTM3 |  |  | 2006 | -0.279 | -0.3628 | No |
| 46 | SNX3 |  |  | 2037 | -0.283 | -0.3689 | No |
| 47 | FAM84B |  |  | 2080 | -0.291 | -0.3783 | No |
| 48 | GATM |  |  | 2082 | -0.292 | -0.3760 | No |
| 49 | ALDH6A1 |  |  | 2091 | -0.294 | -0.3756 | No |
| 50 | LRP4 |  |  | 2111 | -0.300 | -0.3784 | No |
| 51 | RASSF4 |  |  | 2123 | -0.301 | -0.3789 | No |
| 52 | SERPINE2 |  |  | 2128 | -0.302 | -0.3773 | No |
| 53 | TMEM132B |  |  | 2168 | -0.308 | -0.3857 | No |
| 54 | APBB2 |  |  | 2231 | -0.319 | -0.4006 | No |
| 55 | CRYL1 |  |  | 2296 | -0.332 | -0.4160 | No |
| 56 | METTL7A |  |  | 2313 | -0.335 | -0.4176 | No |
| 57 | BMP7 |  |  | 2329 | -0.339 | -0.4189 | No |
| 58 | SEMA6D |  |  | 2377 | -0.349 | -0.4292 | No |
| 59 | SIRPA |  |  | 2398 | -0.353 | -0.4318 | No |
| 60 | ANOS1 |  |  | 2418 | -0.357 | -0.4340 | No |
| 61 | ZFYVE21 |  |  | 2425 | -0.359 | -0.4326 | No |
| 62 | ABAT |  |  | 2438 | -0.365 | -0.4327 | No |
| 63 | B2M |  |  | 2460 | -0.369 | -0.4355 | No |
| 64 | C3orf70 |  |  | 2464 | -0.370 | -0.4330 | No |
| 65 | ITGAV |  |  | 2476 | -0.371 | -0.4329 | No |
| 66 | ABHD3 |  |  | 2487 | -0.375 | -0.4324 | No |
| 67 | PHYHIPL |  |  | 2512 | -0.381 | -0.4359 | No |
| 68 | SLITRK2 |  |  | 2541 | -0.387 | -0.4404 | No |
| 69 | SPRY2 |  |  | 2569 | -0.391 | -0.4447 | No |
| 70 | TSPAN6 |  |  | 2602 | -0.402 | -0.4502 | No |
| 71 | PBXIP1 |  |  | 2725 | -0.431 | -0.4813 | No |
| 72 | PREX1 |  |  | 2727 | -0.432 | -0.4778 | No |
| 73 | DOK5 |  |  | 2799 | -0.457 | -0.4940 | No |
| 74 | RAB31 |  |  | 2806 | -0.459 | -0.4916 | No |
| 75 | PLEKHB1 |  |  | 2810 | -0.461 | -0.4884 | No |
| 76 | TSPAN3 |  |  | 2828 | -0.465 | -0.4891 | No |
| 77 | QKI |  |  | 2844 | -0.474 | -0.4892 | No |
| 78 | TSPAN7 |  |  | 2848 | -0.475 | -0.4858 | No |
| 79 | IFITM3 |  |  | 2851 | -0.477 | -0.4821 | No |
| 80 | SEMA6A |  |  | 2881 | -0.487 | -0.4861 | No |
| 81 | PON2 |  |  | 2884 | -0.490 | -0.4823 | No |
| 82 | TRIL |  |  | 2958 | -0.519 | -0.4985 | No |
| 83 | C1orf122 |  |  | 2977 | -0.528 | -0.4990 | No |
| 84 | LGALS3BP |  |  | 2989 | -0.533 | -0.4974 | No |
| 85 | EFEMP2 |  |  | 3010 | -0.546 | -0.4982 | No |
| 86 | PDLIM3 |  |  | 3011 | -0.546 | -0.4934 | No |
| 87 | ID2 |  |  | 3026 | -0.554 | -0.4924 | No |
| 88 | DCN |  |  | 3027 | -0.554 | -0.4875 | No |
| 89 | FABP5 |  |  | 3079 | -0.577 | -0.4970 | No |
| 90 | MEIS1 |  |  | 3133 | -0.610 | -0.5067 | Yes |
| 91 | GNG11 |  |  | 3137 | -0.612 | -0.5021 | Yes |
| 92 | SPATA6 |  |  | 3143 | -0.614 | -0.4981 | Yes |
| 93 | FADS2 |  |  | 3173 | -0.640 | -0.5007 | Yes |
| 94 | OLFM2 |  |  | 3176 | -0.641 | -0.4955 | Yes |
| 95 | ITM2C |  |  | 3187 | -0.652 | -0.4926 | Yes |
| 96 | AASS |  |  | 3191 | -0.657 | -0.4876 | Yes |
| 97 | STON2 |  |  | 3220 | -0.688 | -0.4895 | Yes |
| 98 | AIF1L |  |  | 3235 | -0.704 | -0.4872 | Yes |
| 99 | ELN |  |  | 3269 | -0.728 | -0.4901 | Yes |
| 100 | SOX9 |  |  | 3280 | -0.739 | -0.4864 | Yes |
| 101 | GNG7 |  |  | 3310 | -0.774 | -0.4878 | Yes |
| 102 | SOX2 |  |  | 3314 | -0.778 | -0.4817 | Yes |
| 103 | MLC1 |  |  | 3326 | -0.793 | -0.4778 | Yes |
| 104 | NCAN |  |  | 3329 | -0.794 | -0.4713 | Yes |
| 105 | HTRA1 |  |  | 3331 | -0.796 | -0.4645 | Yes |
| 106 | TM7SF2 |  |  | 3334 | -0.802 | -0.4579 | Yes |
| 107 | DAG1 |  |  | 3336 | -0.803 | -0.4511 | Yes |
| 108 | DBI |  |  | 3349 | -0.812 | -0.4473 | Yes |
| 109 | DDR1 |  |  | 3350 | -0.814 | -0.4400 | Yes |
| 110 | ETV1 |  |  | 3378 | -0.857 | -0.4401 | Yes |
| 111 | WSCD1 |  |  | 3379 | -0.860 | -0.4324 | Yes |
| 112 | BCHE |  |  | 3405 | -0.909 | -0.4315 | Yes |
| 113 | CD82 |  |  | 3408 | -0.912 | -0.4239 | Yes |
| 114 | PRCP |  |  | 3417 | -0.927 | -0.4180 | Yes |
| 115 | SOX8 |  |  | 3418 | -0.934 | -0.4096 | Yes |
| 116 | MRC2 |  |  | 3439 | -0.981 | -0.4066 | Yes |
| 117 | CD9 |  |  | 3445 | -0.995 | -0.3992 | Yes |
| 118 | SLC1A3 |  |  | 3458 | -1.025 | -0.3935 | Yes |
| 119 | GPRC5B |  |  | 3459 | -1.026 | -0.3843 | Yes |
| 120 | ETV5 |  |  | 3466 | -1.043 | -0.3767 | Yes |
| 121 | TTYH1 |  |  | 3481 | -1.067 | -0.3712 | Yes |
| 122 | TRIB2 |  |  | 3488 | -1.088 | -0.3633 | Yes |
| 123 | LITAF |  |  | 3491 | -1.089 | -0.3541 | Yes |
| 124 | FAT1 |  |  | 3496 | -1.107 | -0.3454 | Yes |
| 125 | MYO10 |  |  | 3516 | -1.170 | -0.3404 | Yes |
| 126 | NPNT |  |  | 3533 | -1.229 | -0.3340 | Yes |
| 127 | KLHDC8A |  |  | 3552 | -1.370 | -0.3270 | Yes |
| 128 | ITPR2 |  |  | 3556 | -1.388 | -0.3155 | Yes |
| 129 | SPRY1 |  |  | 3565 | -1.432 | -0.3050 | Yes |
| 130 | PDPN |  |  | 3580 | -1.590 | -0.2948 | Yes |
| 131 | FAM181B |  |  | 3582 | -1.594 | -0.2809 | Yes |
| 132 | TNC |  |  | 3586 | -1.618 | -0.2673 | Yes |
| 133 | SLC1A2 |  |  | 3589 | -1.634 | -0.2533 | Yes |
| 134 | CLU |  |  | 3601 | -1.801 | -0.2404 | Yes |
| 135 | CXCL14 |  |  | 3605 | -1.867 | -0.2247 | Yes |
| 136 | LRRN3 |  |  | 3606 | -1.871 | -0.2080 | Yes |
| 137 | SCRG1 |  |  | 3613 | -2.165 | -0.1904 | Yes |
| 138 | GPM6B |  |  | 3616 | -2.257 | -0.1708 | Yes |
| 139 | GFAP |  |  | 3619 | -2.337 | -0.1506 | Yes |
| 140 | ID4 |  |  | 3620 | -2.343 | -0.1297 | Yes |
| 141 | S100B |  |  | 3624 | -2.502 | -0.1083 | Yes |
| 142 | C1orf61 |  |  | 3626 | -2.616 | -0.0852 | Yes |
| 143 | FABP7 |  |  | 3629 | -2.788 | -0.0610 | Yes |
| 144 | PTN |  |  | 3632 | -2.826 | -0.0363 | Yes |
| 145 | PTPRZ1 |  |  | 3637 | -4.237 | 0.0003 | Yes |
Table: GSEA details [plain text format]

  

Fig 2: RICHARDS\_ZHONG\_NATURE\_2018\_ASTROCYTES\_UPREG: Random ES distribution      
 Gene set null distribution of ES for **RICHARDS\_ZHONG\_NATURE\_2018\_ASTROCYTES\_UPREG**
